# Molecular Design and Operational Stability: Toward Stable 3D/2D Perovskite Interlayers

**DOI:** 10.1002/advs.202001014

**Published:** 2020-08-16

**Authors:** Sanghyun Paek, Cristina Roldán‐Carmona, Kyung Taek Cho, Marius Franckevičius, Hobeom Kim, Hiroyuke Kanda, Nikita Drigo, Kun‐Han Lin, Mingyuan Pei, Rokas Gegevičius, Hyung Joong Yun, Hoichang Yang, Pascal A. Schouwink, Clémence Corminboeuf, Abdullah M. Asiri, Mohammad Khaja Nazeeruddin

**Affiliations:** ^1^ Group for Molecular Engineering of Functional Materials École Polytechnique Fédérale de Lausanne (EPFL) Sion CH‐1951 Switzerland; ^2^ Department of Chemistry and Energy Engineering Sangmyung University Seoul 03016 Republic of Korea; ^3^ Samsung Electronics Memory Business Flash PA Team Republic of Korea; ^4^ Department of Molecular Compound Physics Center for Physical Sciences and Technology Saulėtekio Avenue 3 Vilnius LT‐10257 Lithuania; ^5^ Laboratory for Computational Molecular Design École Polytechnique Fédérale de Lausanne (EPFL) Lausanne CH‐1015 Switzerland; ^6^ Department of Chemical Engineering Inha University Incheon 22212 Republic of Korea; ^7^ Research Center for Materials Analysis Korea Basic Science Institute (KBSI) Daejeon 34133 Republic of Korea; ^8^ ISIC EPFL Sion CH‐1051 Switzerland; ^9^ Center of Excellence for Advanced Materials Research (CEAMR) King Abdulaziz University P.O. Box 80203 Jeddah 21589 Saudi Arabia

**Keywords:** 3D perovskites, 2D perovskites, bilayer perovskite solar cells, passivation, perfluorobenzylammonium iodide, stable 3D/2D interfaces

## Abstract

Despite organic/inorganic lead halide perovskite solar cells becoming one of the most promising next‐generation photovoltaic materials, instability under heat and light soaking remains unsolved. In this work, a highly hydrophobic cation, perfluorobenzylammonium iodide (5FBzAI), is designed and a 2D perovskite with reinforced intermolecular interactions is engineered, providing improved passivation at the interface that reduces charge recombination and enhances cell stability compared with benchmark 2D systems. Motivated by the strong halogen bond interaction, (5FBzAI)_2_PbI_4_ used as a capping layer aligns in in‐plane crystal orientation, inducing a reproducible increase of ≈60 mV in the *V*_oc_, a twofold improvement compared with its analogous monofluorinated phenylethylammonium iodide (PEAI) recently reported. This endows the system with high power conversion efficiency of 21.65% and extended operational stability after 1100 h of continuous illumination, outlining directions for future work.

## Graphical abstract

2D perovskites are of great importance to increase both the efficiency and stability of perovskite interfaces. Motivated by the stronger halogen bond interaction, (5FBzAI)_2_PbI_4_ used as a capping layer in 3D/2D systems self‐organizes with an in‐plane crystal orientation, inducing a reproducible increase of ≈60 mV in the *V*_oc_, and remarkable operational stability.

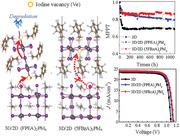

Lead halide perovskite solar cells (PSCs) have demonstrated unprecedented progress in device performance, allowing remarkable power conversion efficiency (PCE) of 25.2% in only one decade. With their unique combination of electrical and optical properties,^[^ ^1^, ^2^, ^3^, ^4^ ^]^ perovskites have the potential to overcome the energy‐intensive manufacturing process associated with silicon photovoltaics while ensuring low‐cost. Yet, hybrid 3D perovskites get easily damaged in the presence of water, oxygen, heat, and UV‐radiation under operating conditions,^[^ ^5^, ^6^ ^]^ hindering large‐scale commercialization. Despite water and oxygen being avoided, a major challenge is to comply with the standard accelerated ageing protocols for long‐term stability, in which thermal and light‐activated decompositions remain problematic.

Among the strategies suggested in literature,^[^ ^7^, ^8^, ^9^ ^]^ 2D perovskites have made a critical entry.^[^ ^8^, ^10^, ^11^, ^12^, ^13^ ^]^ These laminar materials can incorporate larger and less volatile cations, which are highly hydrophobic, endowing the structure with chemical and thermal stability. From the three types of 2D perovskites derived from a parent 3D structure, the most employed in PSCs are the (100)‐oriented systems. They consist of a laminar structure with the general formula A′_2_B*_n_*_−1_PbX_3_ *_n_*_+1_, where *n* planes of [PbX_6_]^−4^ octahedrons are separated by the organic spacers (A′), providing strong versatility in material compositions and properties.^[^ ^14^ ^]^ Thus, the organic layer determines its hydrophobicity,^[^ ^10^, ^15^ ^]^ structural rigidity,^[^ ^16^ ^]^ sheet‐to‐sheet distance,^[^ ^17^ ^]^ and the dielectric properties.^[^ ^18^ ^]^ Nevertheless, low *n* members like A′_2_PbX_4_ exhibit wider optical bandgap, larger exciton binding,^[^ ^10^, ^15^ ^]^ and limited charge transport within certain planes, so that a tradeoff *n* value exists between high efficiency and stability.^[^ ^15^ ^]^ To improve the charge collection, efforts have focused on controlling the orientation of the layered structure toward out‐of‐plane directions. This boosted PCE from 4.02% to 12.52%, with unprecedented device stability.^[^ ^10^ ^]^ However, techniques targeting film morphology and orientation to improve stability without sacrificing efficiency are still critical.

Recently 3D/2D bilayer structures emerged as the most credible path to combine high efficiency and stability. Such configuration exhibits reduced recombination losses and provide a moisture barrier for the 3D material, thus a more durable crystal. At present, benchmark systems for 2D passivation contain phenylethylammonium (PEA) or butyl ammonium salts, and only a few innovative structures demonstrate comparable results.^[^ ^19^, ^20^, ^21^ ^]^ The challenge is to have perfect control over the deposition to form a uniform, conformal 2D layers, with enhanced hydrophobicity and stability. To succeed with rational design, is therefore paramount to understand the mechanisms ruling interfacial interactions and crystal growth. Here, we target the role of chemical substituents at the 2D cations in device performance. Inspired by the outstanding results obtained for the state‐of‐the art PEAI cation,^[^ ^11^ ^]^ and taking advantage of its lower binding energy (*E*_b_) compared to alkyl cations,^[^ ^19^ ^]^ we rationalize their size and hydrophobicity via substitution of fluorine and –CH_2_‐moieties, in order to have a more rigid and super hydrophobic layered structure. We have investigated a monofluorinated PEAI cation (FPEAI) recently reported in the literature,^[^ ^14^, ^22^ ^]^ compared with a novel pentafluorobenzyl ammonium (5FBzAI) cation, which contains a methylene bridge that confers increased rigidity, and compensates the strong dispersion forces induced by the five fluorine units. Given the increased number of F sites and reduced length of 5FBzAI, it strongly interacts with the 3D interface via halogen bond passivation. Our results demonstrate that the organic counterpart in the 2D system dictates the interfacial chemistry at the 3D/2D bilayer, promoting strong variations in 2D electronic properties, crystal packing, and orientation. Importantly, such differences do not necessarily impact the power conversion efficiency, over 21% in all cases, but has a remarkable effect in the long‐term device stability. Consequently, high PCE values up to 21.65%, with 60 mV increase in *V*_oc_, and improved photostability over 1100 h are observed for the new (5FBzAI)_2_PbI_4_ material, thanks to strong passivation via in‐plane crystal growth orientation. Our results emphasize the complex relationships between the device optoelectronics and the intermolecular interactions at the 2D level, evidencing for the first time a possible link between the cationic structure, the 3D/2D interfacial interactions, and the long‐term device stability.

The device configuration employed in this study is based on n‐i‐p architecture containing fluorine‐doped tin oxide (FTO)/compact‐TiO_2_/mesoporous‐TiO_2_/compact‐SnO_2_/3D/2D/Spiro‐OMeTAD/Au, where the 3D absorber consists on an optimized lead excess Cs_0.1_(FA_0.86_MA_0.14_)_0.9_Pb(I_0.86_Br_0.14_)_3_ formulation fabricated by one‐step antisolvent dropping method (see the Experimental Section for further details).^[^ ^23^, ^24^ ^]^ **Figure** **1**a shows the molecular structure of the cations employed for the 2D systems. To identify the effect of F, we chose different ammonium salts, FPEAI and 5FBzAI, that were expected to have similar size once incorporated to the 2D crystal.^[^ ^25^, ^26^ ^]^ Taking PEAI as the reference, we have gradually incorporated highly electronegative fluorine units, from one to five, in order to i) confer super hydrophobicity to the system, ii) identify the effect in the crystal structure, and iii) analyze its influence on the optoelectronic properties. The 3D/2D bilayer architecture was prepared by directly spin‐casting 100 mL of the corresponding benzyl derivative cation in isopropanol on the 3D absorber, followed by a short annealing process at 100 °C for 10 min. The effect of the different cations on the optoelectronic properties was analyzed by ultraviolet–visible (UV–vis) and ultraviolet photoelectron spectroscopy (UPS). Figure 1b shows the UV–vis absorption spectra of a typical 3D perovskite, along with the 3D/2D bilayer containing FPEA_2_PbI_4_ and 5FBzA_2_PbI_4_ perovskites. The absorption corresponding to the pure 2D systems is also included as reference, showing sharp and intense peaks, assigned to the excitonic 2D feature, followed by a continuous absorption related to direct band‐to‐band transitions. Notably, the peak position and band edges between both 2D perovskites are strongly shifted, suggesting a clear variation on their electronic structure induced by the organic substituents.^[^ ^18^ ^]^ The 2D bandgap energy (*E*_g_), estimated through Tauc plots analysis (see inset in Figure 1c), gives values of 2.34 and 2.52 eV for (FPEA)_2_PbI_4_ and (5FBzA)_2_PbI_4_, respectively. Conversely, no significant changes in absorption coefficient and spectrum shape are detected for the 3D perovskite after the incorporation of the 2D interlayer. The bilayer films preserve high and broad absorption in the complete visible region, as for the pristine 3D material, and *E*_g_ values of ≈1.6 eV (slightly red shifted in line with previous PEAI and FPEAI perovskite reports).^[^ ^14^, ^27^ ^]^ The 2D material is only detected in the high‐energy region by a gradual increase in absorption below 550 nm. We used UPS to investigate the electronic structure of the 3D and 3D/2D bilayer systems. The results, plotted in Figure 1d, revealed a shift on the valence band onset away from the Fermi level (by 0.3 eV) for the 3D/2D system employing one F substituted cation, while retaining the main valence band (VB) features typically observed in the 3D. On the contrary, the introduction of five‐fluorinated 5FBzAI cation conducts a solid change, shifting the VB onset back toward the Fermi level, closer to the pristine 3D material. Note that, despite the organic cation does not generally affect electronic properties, the intermolecular packing in the layered structure is highly affected by the chemical substituents, which can alter the noncovalent R···PbI interactions with the 2D inorganic network, thus tuning the inorganic Pb/I orbital overlap.^[^ ^19^ ^]^ The energy level diagram obtained for both 3D/2D systems, combining the Tauc plot and UPS results, is illustrated in Figure 1d (inset). Concomitantly with the VB changes, due to the high‐energy bandgap of 2D perovskites (see Figure 1c), an energy band offset appears at the 3D perovskite surface, which ensures electron blocking and boosts electron transfer toward the opposite side, at the electron‐transporting layer (ETL).

**Figure 1:**
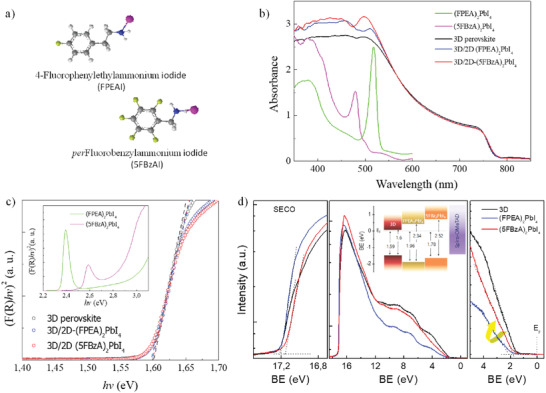
UPS and optical characterization. a) Schematic of the two ammonium salts, FPEAI and 5FBzAI, employed in the 2D perovskites. b) UV–vis spectra of the 3D, 2D, and 3D/2D bilayer systems. c) Tauc plot of the layers shown in (b). d) UPS spectra of 3D and 3D/2D systems showing the secondary cut‐off region (left) and onset region (right) for each film. Inset: energy level diagram of the 3D/2D systems.

We also analyzed the film morphology and crystal structure using scanning electron microscopy (SEM) and X‐ray diffraction (XRD) measurements (see the Experimental Section for details). **Figure** **2** shows the top view of SEM images corresponding to 3D perovskite, before and after the 2D treatment. The pristine perovskite exhibits the typical multicrystaline pinhole‐free morphology homogeneously distributed through the substrate, with large grain crystals of ≈400 nm. After the 2D treatment the surface texture changes, becoming flatter and covered by a thin overlayer of ≈30 nm (see Figure 2c–f). A better surface coverage and homogeneity is also detected for 5FBzAI (see Figure 2e).^[^ ^28^ ^]^ We investigated the crystal structure of such bilayer by XRD measurements, as presented in Figure 2g. The pristine 3D perovskite exhibits the typical XRD pattern reported for the mixed cationic formulation, together with small additional peaks corresponding to *δ*‐FAPbI_3_ (11.6°) and PbI_2_ (12.7°) phases.^[^ ^29^, ^30^ ^]^ Note that PbI_2_ excess disappears after the 2D surface treatment, and additional diffraction peaks at low angles reveal the formation of concomitant 2D systems (Figure 2h). To identify these materials, we prepared films of pure FPEA_2_PbI_4_ and 5FBzA_2_PbI_4_ (included in Figure 2g as reference), and single crystals of 5FBzA_2_PbI_4_ (see crystallographic properties in Table S1 in the Supporting Information). Interestingly, a notable shift to lower angles in 5FBzA_2_PbI_4_ suggests an expansion of the unit cell volume compared to FPEA_2_PbI_4_ (see zoom of (100) lattice plane in the inset)_._^[^ ^14^ ^]^ According to Bragg's equation, the *d* spacing between 2D planes shifts from 16.51 Å (5.35°) to 17.66 Å (5.0°), respectively. This implies that, although 5FBzAI contains one less methylene‐bridge compared to FPEAI, the dispersion forces promoted by F groups and the rigidity introduced via removal of methylene unit results in a more expanded packing (as illustrated in **Figure** **3**a,b). This indeed causes a shift in the 2D crystal structure from monoclinic^[^ ^31^ ^]^ to a more symmetric orthorhombic system. In addition, 3D/2D containing 5FBzAI presents more than twofold increase in diffraction intensity for a similar layer thickness, suggesting either improved crystallinity or a different preferred orientation for the 2D portion.

**Figure 2:**
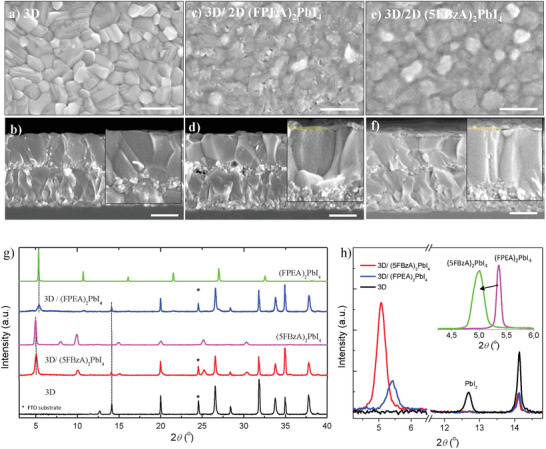
Morphological analysis and XRD characterization of 3D/2D systems. a,c,e) Top‐surface SEM images of the 3D/2D bilayer structures containing FPEAI and 5FBzAI cations. b,d,f) Cross‐sectional SEM pictures of the devices shown in (b), (d), and (f). Scale bar in all images equals to 500 nm. g) Powder XRD patterns obtained from thin films of 3D and 3D/2D systems deposited on FTO/TiO_2_ substrates. Thin films of pure 2D perovskites deposited on glass are also included for reference. h) Magnification of the XRD peaks observed at 2*θ* < 15°. Inset: XRD peak at (100) plane in 2D.

**Figure 3:**
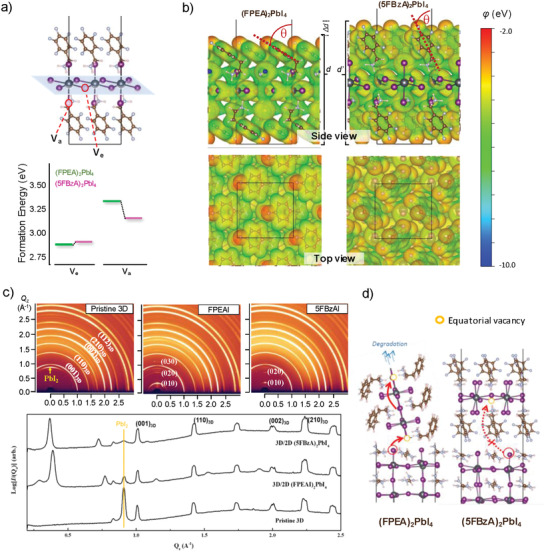
DFT calculations and crystal orientation analysis. a) Formation energy in eV of neutral iodine vacancy in 2D perovskites containing FPEAI and 5BzAI cations. b) Electrostatic potential at the surface of 2D perovskites shown in (a). c) 2D‐GIWAXS plots and azimuthally integrated intensity of pristine 3D and 3D/2D films prepared from FPEAI and 5FBzAI cations. d) Schematic representation of ion migration for 3D/2D bilayers with different crystal orientations.

To evaluate the role of 5FBzAI cation at the 3D/2D interface, we performed density functional theory (DFT) computations based on the defect model, and analyzed the formation energy of neutral iodine vacancy of both type of perovskites, FPEA_2_PbI_4_ and 5FBzA_2_PbI_4._ The formation energy of iodine vacancies for both equatorial (*V*_e_) and apical (*V*_a_) positions in the 2D, shown in Figure 3a, revealed increased energy for the later, suggesting that iodine vacancies form in both systems preferably at equatorial sites. Interestingly, the binding energy, calculated in terms of chemical interaction between the 2D and 3D (see Note S1 in the Supporting Information), suggests a much stronger interaction for 5FPEA cation, presumably due to the halogen–halogen interaction between F and I. In addition, the crystal packing and electrostatic potential (*φ*) maps, depicted in Figure 3b, reveal an overall increase of surface *φ* dictated by the number F atoms. This implies that 5FBzA_2_PbI_4_ becomes less attractive to negative charge, and thus it behaves as a more effective electron barrier at the perovskite/hole transporting material (HTM) interface. Noteworthily, this is only true given a 2D crystal orientation parallel to the interface. We investigated the structural properties by recording grazing incidence X‐ray diffraction (GIXD) and azimuthally integrated intensity from 2D and 3D/2D systems. As observed in Figure 3c, the 3D material was randomly oriented in all cases, as determined by Debye–Scherrer rings. An arbitrary distribution was also detected after depositing FPEAI, suggesting an irregular arrangement of FPEA_2_PbI_4_ crystals on top of the 3D layer. Similar results have been recently reported for FPEA_2_PbI_4_ system, supporting these observations.^[^ ^22^ ^]^ However, the novel 5FBzA_2_PbI_4_ is strongly oriented, with 2D layers arranged ideally parallel to the 3D surface. This implies that the interfacial interactions, induced by the substituents at the organic cations, may rule the crystal growth of the perovskite interlayers, inducing a parallel or random crystal orientation of the 2D component. Notably, a random orientation implies the absence of a continuous crystal plane covering the surface, which might induce a high portion of uncoordinated species at the 3D/2D interface, where crystal defects are easily formed. Because the activation energy for defect diffusion is lower at the interface than in the bulk, and in the 2D it preferably occurs within the inorganic [PbI_6_]^−4^ planes, a 3D/2D bilayer containing irregularly grown FPEA_2_PbI_4_ crystals will be more prone to ion migration.^[^ ^32^ ^]^ Given the strong correlation between ion migration and degradation,^[^ ^18^ ^]^ such a different orientation will certainly impact the 3D/2D optoelectronic behavior in long‐term, as later demonstrated in this manuscript. The schematic of the proposed ion migration mechanism is illustrated in Figure 3d.

To test the applicability of 3D/(5FBzA)_2_PbI_4_ system and its impact on device performance, we embodied the bilayer architectures into PSCs. Reference cells using triple cation 3D perovskite and Spiro‐OMeTAD as HTM were prepared for comparison. **Figure** **4**a shows the champion cells obtained for each condition, along with the initial maximum power point (mpp) tracking located in the inset. As observed, the device efficiency obtained for the reference 3D material showed a high PCE of 20.22%, with an open‐circuit voltage (*V*_oc_) of 1.09 V, short‐circuit current density (*J*_sc_) of 23.48 mA cm^−2^, and a fill factor (FF) of 0.79. After incorporating the 3D/2D bilayer containing FPEAI, the efficiency increased over 21.31%, showing *V*_oc_ values of 1.13 V for the champion cell (*J*_sc_ of 23.88 mA cm^−2^ and FF of 0.79). Note that despite *J*_sc_ was slightly improved, the main factor contributing to the increased efficiency was the *V*_oc_, with 40 mV higher than the reference. Interestingly, such efficiency was further exceeded when employing 5FBzAI as cation, which led to a remarkable and reproducible increase of ≈60 mV, in agreement with the improved passivation expected from an in‐plane crystal alignment. The champion cell exhibited 1.15 V, with a *J*_sc_ value of 24.14 mA cm^−2^, and a FF of 0.78, leading to an improved PCE of 21.65%. As a remark, very small hysteresis between reverse and forward scans was detected (see Figure S1 in the Supporting Information). Importantly, the initial mpp tracking under 1 sun illumination demonstrates PCE drop of 5% using 3D/FPEA_2_PbI_4_, while that using 5FBzA_2_PbI_4_ remains stabilized at 21.37%. The incident photon‐to‐electron conversion efficiency (IPCE) of each PSC is also presented in Figure 4b. Very similar spectra are obtained for the three systems, with only slight increased photoresponse for the bilayer 3D/2D structures, leading to an integrated current density (*J*_int_) of 22.82, 23.18, and 23.35 mA cm^−2^ for pristine 3D, 3D/2D‐(FPEA)_2_PbI_4_, and 3D/2D‐(5FBzA)_2_PbI_4_, respectively. The device statistics obtained from 50 cells are presented in Figure 4c and Figure S2 in the Supporting Information, where the increased efficiency attributed to a remarkable high *V*_oc_ for 5FBzAI can be also appreciated.

**Figure 4:**
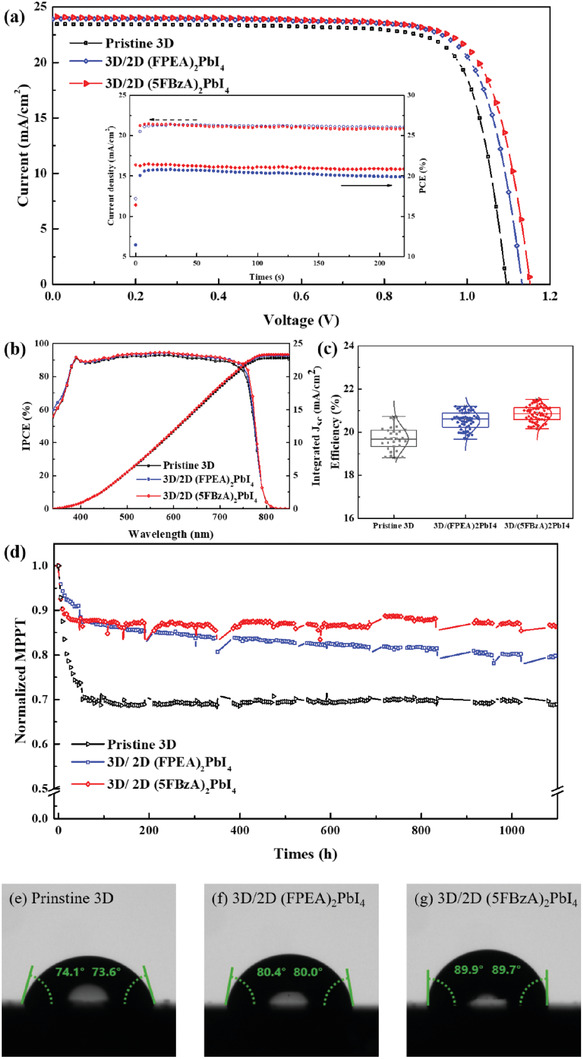
Device photovoltaic performance and operational stability. a) *J*–*V* curves of the champion cells employing 3D, and 3D/2D bilayer systems containing FPEAI and 5FBzAI cations under AM1.5 illumination. Inset: stabilized PCEs at mpp tracking during 220 s. b) IPCE (left) and integrated photocurrent (right) from cells presented in (a). c) Histograms showing the PCE values obtained from 60 devices. d) Photostability test from maximum power point tracking under 1100 h continuous illumination of full sun light in Ar. The contact angle measurements performed on the e) pristine 3D, f) 3D/2D (FPEA)_2_PbI_4_, and g) 3D/2D (5FBzA)_2_PbI_4_ surfaces.

To gain insight into the device stability, nonencapsulated 3D/2D bilayers were analyzed under continuous light illumination (AM1.5 G) during 1100 h at 25 °C. The cells were kept at mpp tracking, and the current–voltage curves were recorded every 3 h automatically. The results, presented in Figure 4d, revealed very strong influence of the 2D system employed, which in all cases had a fast initial decay (<50 h), followed by a gradual stabilization of the performance. Such fast burn‐in, recently associated to Spiro‐OMeTAD and gold interpenetration,^[^ ^33^ ^]^ strongly affects the cells with pristine 3D material, reducing by 30% its initial PCE. Among the 3D/2D systems, the best stability is observed for that containing 5FBzAI, which retained 86% its initial efficiency after 1100 h of test. Compared with FPEAI, such stabilization is mostly attributed to a more stable *V*_oc_ and FF (Figure S3, Supporting Information), plausibly associated to enhanced defect passivation and more stable 3D/2D interface. Stability tests performed in dark, under controlled humidity showed similar results (see Figure S4 in the Supporting Information), providing evidence of the beneficial effect of 5FBzAI. This was also verified by performing contact angle measurements to investigate the changes in hydrophobicity induced by the cation (see Figure 4e–g). As expected, the water affinity of the surface decreased from mono‐ to pentafluoro substitution. The 3D/(5FBzA)_2_PbI_4_ bilayer showed the highest hydrophobicity (contact angle ≈90°), and a greater photovoltaic stability under humid environments (see Figure S4 in the Supporting Information).

The evolution in the optical properties for 3D/2D bilayers over 2000 h is also presented in Figure S5 in the Supporting Information. Regardless of the perovskite composition, the absorption and PL spectra of the as‐deposited films were very similar, showing absorption onset and PL peak close to 760 nm. Meanwhile, when exposed to aging, the pristine 3D undergoes degradation, manifested by the decrease of absorption intensity and PL blue‐shifting. In contrast, none of the 3D/2D bilayers revealed any changes, confirming the surface preservation against external light stimuli. Interestingly, this implies that the efficiency losses, which are most pronounced within the 50 h of devices testing (see Figure 4d), can be hardly related to the same processes that cause degradation of the perovskite films. We additionally performed time‐resolved photoluminescence (trPL) measurements on freshly prepared and aged perovskite films to further understand the causes behind the observed stability trends. **Figure** **5**a–c shows the photoluminescence decay kinetics of the pristine 3D, 3D/2D (FPEA)_2_PbI_4_ and 3D/2D (5FBzA)_2_PbI_4_ perovskites measured at different aging times. We used two exponential decay components to accurately fit PL decays with the parameters given in Table S2 in the Supporting Information. According to the trPL measurements, perovskite films show fast and slow decay components attributed to the electron trapping and Shockley Read Hall (SHR) recombination, respectively.^[^ ^34^, ^35^ ^]^ The slow PL decay component (*τ* ≈ 1 µs), common to all as‐deposited perovskite films, suggests a high film quality regardless of its composition. However, differences become more evident after aging, and a strong crystal deterioration promoted by the light exposure is detected in the reference 3D material. A high density of trap states, suggested by more than five times reduced PL lifetime serves as recombination sites, reducing the free‐path distance for charge carriers. Meanwhile, PL decay kinetics after FPEAI and 5FBzAI treatments is less affected, confirming the effective passivation and stability induced by both cations.

**Figure 5:**
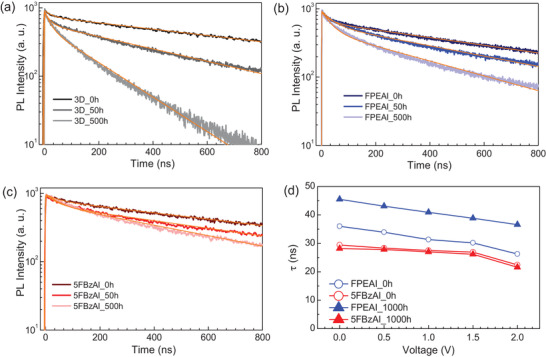
Voltage‐dependent trPL measurements. a–c) Photoluminescence decay kinetics of the as‐deposited and aged pristine 3D, 3D/2D (FPEA)_2_PbI_4_, and 3D/2D (5FBzA)_2_PbI_4_ perovskite films. d) Electric field‐dependent average photoluminescence lifetimes of the FPEAI (blue) and 5FBzAI (red) treated perovskite solar cells before the aging (empty circles) and after 1000 h of aging (filled triangles).

We also performed voltage‐dependent transient photocurrent measurements to compare the charge carrier extraction abilities in the pristine 3D and 3D/2D treated perovskite devices. Photocurrent decay kinetics obtained under various DC voltages of the fresh and aged devices is shown in Figure S6 in the Supporting Information. The photocurrent decay lifetimes calculated using a single exponential decay fit are summarized in Figure S7 in the Supporting Information. After sample excitation with a short light pulse, the concentration of photogenerated charge decreases due to multiple processes such as carrier trapping, recombination or extraction on the electrodes. Considering that we use low light intensity for sample excitation, the influence of bimolecular recombination could be ignored.^[^ ^36^ ^]^ Consequently, the photocurrent decrease under no external voltage is mainly governed by SHR recombination or interface carrier trapping, while under increased bias voltage, a more efficient charge carrier extraction leads to faster photocurrent decay. We note here that the decay time of the fresh PSCs at 0 V bias voltage is very similar, however it becomes faster for aged solar cells. Thus, it might be associated with the deterioration of the material quality due to light induced degradation. The most pronounced change in photocurrent lifetimes between fresh and aged cells occurs for FPEAI treated perovskites, whereas it remains very similar in the samples employing 5FBzAI cation, suggesting their improved resistance against photodegradation. Notably, upon increasing the applied voltage, the photocurrent decay kinetics of all samples becomes faster, and this acceleration might be governed by the faster carrier extraction. In addition, the dynamics of the photocurrent in FPEAI treated devices has little dependence on the applied field, which indicates a poorer extraction of charge carriers into the electrodes compared to pristine 3D and 5FBzAI containing devices. Besides the influence of aging is most pronounced in the reference and FPEAI treated solar cells, meanwhile the photocurrent decay kinetics in 5FBzAI‐based solar cells remains almost unchanged. It can be concluded that the 5FBzAI pretreatment positively affects perovskite film quality in the aging process, and the resulting device has better carrier extraction abilities due to effective surface passivation.

To further understand the role of cations on the charge carrier transport properties, we additionally performed voltage‐dependent trPL measurements. Because the PL dynamics of the complete cell is governed by multiple processes that determine its complex kinetics,^[^ ^35^ ^]^ for the simplicity we used average PL lifetimes calculated from double exponential fits: $\bar{\tau }=\sum_{i=1}^{2}A_{i}{\tau_{i}}^{2}/\sum_{i=1}^{2}A_{i}\tau_{i}$. We believe that this simplification will facilitate analysis without the risk of ignoring the underlying processes, since the perovskite contact with TiO_2_ and Spiro‐OMeTAD is identical and thus, the PL relaxation is mainly determined by the quality of the absorber. Figure 5d shows the averaged trPL lifetimes of FPEAI and 5FBzAI treated cells under applied external voltages ranging from 0 to 2 V. Note that in the case of complete devices, the PL decays more rapidly due to efficient carrier extraction from the absorber to the hole and electron transporting layers. In particular, before the aging and under zero applied voltage, PL decays with very similar lifetimes for both systems (≈36 ns for FPEAI‐ and ≈30 ns for 5FBzAI‐based devices). Yet, the influence of the treatment becomes more obvious with time. The average PL lifetime for aged 3D/(FPEA)_2_PbI_4_ bilayer increases by about ten nanoseconds compared to the fresh ones; but it remains unchanged for 5FBzAI‐based PSCs. The weak voltage dependent PL quenching in 5FBzAI‐based solar cells indicates that electric field in perovskite layer is screened and thus hole transport through the interface is dominated by diffusion, rather than drift and thus the additional field do not change the hole transfer efficiency. Whereas electric‐field dependent PL quenching for FPEA‐based devices suggest that additional field is required to accelerate carrier transfer through the interface. This implies reduced charge transfer efficiency at the 3D/(FPEA)_2_PbI_4_/Spiro‐OMeTAD interface, thus reduced hole transfer yield. Note that the aging most likely leads to a loss of ohmic contact between 3D/2D bilayers containing FPEAI and Spiro‐OMeTAD, which also may cause a slight decrease in the *V*_oc_, as previously suggested by Figure S3 in the Supporting Information. This allows us to conclude that the time‐dependent *V*_oc_ for FPEAI treated devices is undoubtedly related with the interface degradation rather than increase in trap density that can act as nonradiative recombination centers for the charge carriers.

In summary, based on DFT calculations and voltage‐dependent trPL measurements we have demonstrated that the intermolecular interactions and crystal packing induced by the 2D organic cation rule the interfacial properties at the perovskite/HTM, becoming decisive for a highly efficient and stable 3D/2D interface. We have performed a rational design of a novel fluoro‐substituted 2D material, 5FBzAI_2_PbI_4,_ which strongly interacts with the 3D perovskite, inducing a highly in‐plane oriented growth of the crystals while preserving excellent hole transfer. Compared to the pristine perovskite, a reproducible increase of 60 mV in the *V*_oc_ is obtained using 5FBzAI cation, and a twofold rise compared to the benchmark FPEAI_2_PbI_4_. Our results emphasizes the importance of a rational design of the 2D material at the molecular level, demonstrating for the first time a direct relation between the organic cation, via chemical substituents, and the solar cell operational stability. Therefore, studies targeting rational perturbations on the organic component while preserving the inorganic layer are highly desirable, and may open up a plethora of novel molecularly designed highly stable 2D interlayers.

## Experimental Section

##### Materials

PbI_2_ and PbBr_2_ were purchased from TCI, CsI 99.998% grade ultradry from abcr, while FAI and MABr were bought from GreatCell solar. FPEAI was synthesized by reacting 2‐(4‐fluorophenyl)ethylamine with HI as reported.^[^ ^14^ ^]^ Perflourobenzyl amine was synthesized by reducing pentafluorobenzonitrile with BBr_3_.^[^ ^25^ ^]^ 5FBzAI was diluted in 10 mL of anhydrous ethanol and 1.5 eq. of HI was reacted in 50 mL at 0 °C for 1 h stirring. The precipitate was recovered by evaporating the solvent at 50 °C for 1 h. 5FBzAI was saturated in ethanol, recrystallized from diethyl ether, and finally dried at 60 °C for 24 h.

##### Crystal Growth Procedure

PbI_2_ (1 mol eq.) was dissolved in concentrated aqueous HI (57 wt%) at RT followed by addition of aqueous solution of 5FBzAI (2 mol eq.). An aliquot of the resulting mixture was further transferred onto a watch glass and allowed to evaporate at RT to obtain crystalline solids suitable for the single‐crystal XRD analysis.

##### Solar Cell Fabrication

FTO (Nippon Sheet) glass was cleaned by sonicating in a 2% Hellmanex solution, water, acetone, and ethanol, followed by a 15 min UV‐ozone treatment. 30 nm TiO_2_ was deposited by spray pyrolysis at 450 °C from titanium diisopropoxide bis(acetylacetonate) (TAA; Sigma‐Aldrich) in isopropanol. Mesoporous TiO_2_ films were prepared using diluted TiO_2_ paste (Dyesol 30 NR‐D) by spin‐coating and later sintering at 500 °C for 30 min. SnO_2_ layer was prepared by spin‐coating 0.1 m of SnCl_4_ aqueous solution, sintered at 180 °C for 1 h. The perovskite solution was prepared by dissolving PbI_2_ (1.15 m), FAI (1.10 m), PbBr_2_ (0.2 m), and MABr (0.2 m) in an anhydrous solvent DMF:DMSO = 4:1 (volume ratio). 1.15 m solution of CsPbI_3_ was also made using the same volume ratio of DMF:DMSO. Finally (FAPbI_3_)_0.86_(MAPbBr_3_)_0.14_ and CsPbI_3_ solutions were mixed in the ratio of 10 vol%, and spin‐coated at 1000 rpm for 12 s, followed by 5000 rpm for 30 s. Then, trifluorotoluene (110 µL) was dropped 15 s prior to complete the process, and films were annealed at 100 °C for 90 min. 2D perovskite was then deposited by spin‐casting 100 mL of FPEAI and 5FBzAI at 4000 rpm in isopropanol and annealed at 100 °C for 10 min. Spiro‐OMeTAD was spin‐coated at 4000 rpm for 25 s from 70 × 10^−3^ m in chlorobenzene mixed with *tert*‐butylpyridine (*t*BP), tris(2‐(1*H*‐pyrazol‐1‐yl)‐4‐*tert*‐butylpyridine)‐cobalt(III) (FK209), and tris(bis(trifluoromethylsulfonyl)imide) (Li‐TFSI) (330 mol% *t*BP, 50 mol% Li‐TFSI from 1.8 m stock solution, and 3 mol% FK209 from 0.25 m stock solution, in acetonitrile). 70 nm of Au was deposited as electrode.

##### Thin Film Characterization

Absorbance spectra were taken with UV/vis/NIR spectrophotometer (PerkinElmer Lamda). XRD analysis in an angle range of 2*θ* = 2°–40° was performed using a Bruker D8 Advance diffractometer. Also, synchrotron‐based 2D GIXD was performed on the films at the Pohang Accelerator Laboratory, 6D and 9A beamlines.^[^ ^37^ ^]^ A sample was mounted on a two‐axis goniometer on top of an *x*–*z* stage, and the scattering intensity was measured using a 2D CCD detector. High‐resolution SEM images were obtained on a ZEISS Merlin at an accelerating voltage of 5 kV. Steady state fluorescence spectra were recorded with fluorescence spectrometer F900 (Edinburgh Instruments Co., UK); PL decays were measured using Edinburgh Instruments time‐correlated single photon counting fluorescence spectrometer F900. Semiconductor diode laser EPL‐470 emitting 72 ps pulses at 470 nm was utilized in transient measurements for sample excitation (pulse repetition rate 500 kHz (2 µs), time resolution of several hundreds of picoseconds by applying apparatus function deconvolution).

##### Device Characterization

AM1.5 simulated light source was connected with a source meter (Keithley 2400). Light intensity was calibrated with NREL certified KG5 filtered Si reference diode. *J*–*V* curves were obtained using black mask with 0.16 cm^2^ active area (50 mV s^−1^). IPCE spectra were obtained using ORIEL, IQE 200B instrument. For stability tests, unsealed solar cells under Ar were subjected to constant light soaking at 100 mW cm^−2^, maintained at maximum power output using electronic control at a room temperature, making automatic *J*–*V* measurements every 3 h (intensity verified with a reference Si‐photodiode). Transient photocurrent measurements were performed with an Agilent Technologies DS05054A oscilloscope using 50 Ω input resistor and a Tektronix AFG 3101 function generator. To minimize electric field induced ionic movement, each electrical pulse was followed by 2–4 ms relaxation time at zero applied field. Samples were excited by radiation of the optical parametric amplifier Topas‐C (Light Conversion Ltd.) pumped by femtosecond Ti:sapphire laser Integra‐C from Quantronix Inc. generating 130 fs duration pulses at 430 Hz repetition rate. Collinear optical parametric amplifier TOPAS‐C was used for the generation of the excitation pulse emitting at 500 nm.

##### Computational Calculations

DFT with Perdew–Burke–Ernzerhof (PBE)^[^ ^38^, ^39^ ^]^ functional along with D3BJ dispersion correction was used,^[^ ^40^ ^]^ as implemented in the Vienna ab initio simulation package.^[^ ^41^ ^]^ Starting from experimental 2D crystal structures (FPEAI^[^ ^31^ ^]^ and 5FBzAI), they were optimized until the residual forces on the constituent atoms became less than 0.02 eV A˚^−1^, while keeping the lattice parameters fixed. The valence electron wave functions were expanded in plane‐wave basis sets and the projector augmented wave (PAW) method was used to describe the core‐electron interactions.^[^ ^42^ ^]^ The plane‐wave cutoff energy was set to be 550 eV and the Brillouin zone samplings were performed using a 3 × 3 × 1 *k*‐point grid in the Monkhorst–Pack scheme. The formation energy of neutral iodine vacancy was computed as following

(1) $$\begin{array}{c} E\_F=E\_\left(2D+V\_I\right)+\left(1/2E\right)\_\left(I\_2\right)-E\_2D \end{array}$$

*E*_F is the formation energy of neutral iodine vacancy, *E*_(2D+*V*_I) is the total electronic energy of the 2D structure with one neutral iodine vacancy, *E*_(I_2) is the total electronic energy of iodine molecule, and *E*_2D is the total electronic energy of the 2D structure. The 2D electrostatic potential color maps were visualized using VESTA.^[^ ^43^ ^]^

The 3D/2D stacks were built with the 3D perovskite approximated with FAPbI_3_ terminated with FA cation layer. The lattice constants of the 2D structures were adopted to that of FAPbI_3_, where the lattice mismatch and the binding energy were evaluated (see Note S1 in the Supporting Information).

## Conflict of Interest

The authors declare no conflict of interest.

## Author Contributions

S.P., K.T.C., C.R‐C., and M.K.N. conceived the ideas and S.P. and C.R‐C. wrote the manuscript. S.P. prepared, characterized, and fabricated the films or cells. M.P. and H.Y. performed GIWAXS experiments. H.J.Y. and H.K. performed UPS for surface analysis. M.F. and R.G. performed trPL measurements. C.R‐C. performed scanning electron microscopy and XRD analysis. N.D. and P.A.S. grew and measured the crystals. H.K. and A.M.A. estimated the long‐term stability of the devices. K.‐H.L. and C.C. performed DFT calculation. All authors discussed the results.

## Supporting information

Supporting Information
