# Supplementary material for: Molecular Design and Operational Stability: Toward Stable 3D/2D Perovskite Interlayers
Source: Adv Sci (Weinh). 2020 Aug 16;7(19):2001014. doi: 10.1002/advs.202001014 (PMC7539205; doi:10.1002/advs.202001014)
Supplement: Supplementary file 1 — Supporting Information [file ADVS-7-2001014-s001.pdf]

© 2020 Wiley-VCH GmbH

ADVANCED SCIENCE
Open Access

Supporting Information

for *Adv. Sci.,* DOI: 10.1002/advs.202001014

Molecular Design and Operational Stability: Toward Stable 3D/2D Perovskite Interlayers

*Sanghyun Paek, Cristina Roldán-Carmona, Kyung Taek Cho, Marius Franckevi ̌cius, Hobeom Kim, Hiroyuke Kanda, Nikita Drigo, Kun-Han Lin, Mingyuan Pei, Rokas Gegevi ̌cius, Hyung Joong Yun, Hoichang Yang, Pascal A. Schouwink, Clémence Corminboeuf, Abdullah M. Asiri, and Mohammad Khaja Nazeeruddin**

Molecular design and operational stability: towards stable 3D/2D perovskite interlayers

Sanghyun Paek,[1,9] Cristina Roldán-Carmona,[1] Kyung Taek Cho,[1,8] Marius Franckevičius,[2] Hobeom Kim,[1] Hiroyuke Kanda,[1] Nikita Drigo,[1] Kun-Han Lin,[3] Mingyuan Pei,[4] Rokas Gegevičius,[2] Hyung Joong Yun,[5] Hoichang Yang,[4] Pascal A. Schouwink,[6] Clémence Corminboeuf,[3] Abdullah M. Asiri,[7] and Mohammad Khaja Nazeeruddin[1,]*

Dr. S. Paek, Dr. C. Roldan Carmona, Dr. K. T. Cho, Dr. H. Kim, Dr. Hiroyuke Kanda, Nikita Drigo, and Prof. Md. K. Nazeeruddin. Group for Molecular Engineering of Functional Materials, École Polytechnique Fédérale de Lausanne (EPFL), CH-1951 Sion, Switzerland
E-mail: (mdkhaja.nazeeruddi@epfl.ch)

Dr. M. Franckevičius, R. Gegevičius. Department of Molecular Compound Physics, Center for Physical Sciences and Technology, Saulėtekio Avenue 3, LT-10257 Vilnius, Lithuania.

K. -H. Lin, Prof. C. Corminboeuf. Laboratory for Computational Molecular Design, École Polytechnique Fédérale de Lausanne (EPFL), CH-1015 Lausanne, Switzerland

M. Pei, Prof. H. Yang. Department of Chemical Engineering, Inha University, Incheon 22212, Republic of Korea

Dr. H. J. Yun. Advance Nano Research Group, Korea Basic Science Institute (KBSI), Daejeon 34133, Republic of Korea.

Dr. P. A. Schouwink ISIC, EPFL, CH-1051 Sion, Switzerland

Prof. Abdullah M. Asiri, Center of Excellence for Advanced Materials Research (CEAMR), King Abdulaziz University, P.O. Box 80203, 21589 Jeddah, Saudi Arabia.

Dr. K. T. Cho. Samsung electronics. Memory business. Flash PA team.

Dr. S. Paek. Department of Chemistry and Energy Engineering, Sangmyung University, Seoul 03016, Republic of Korea

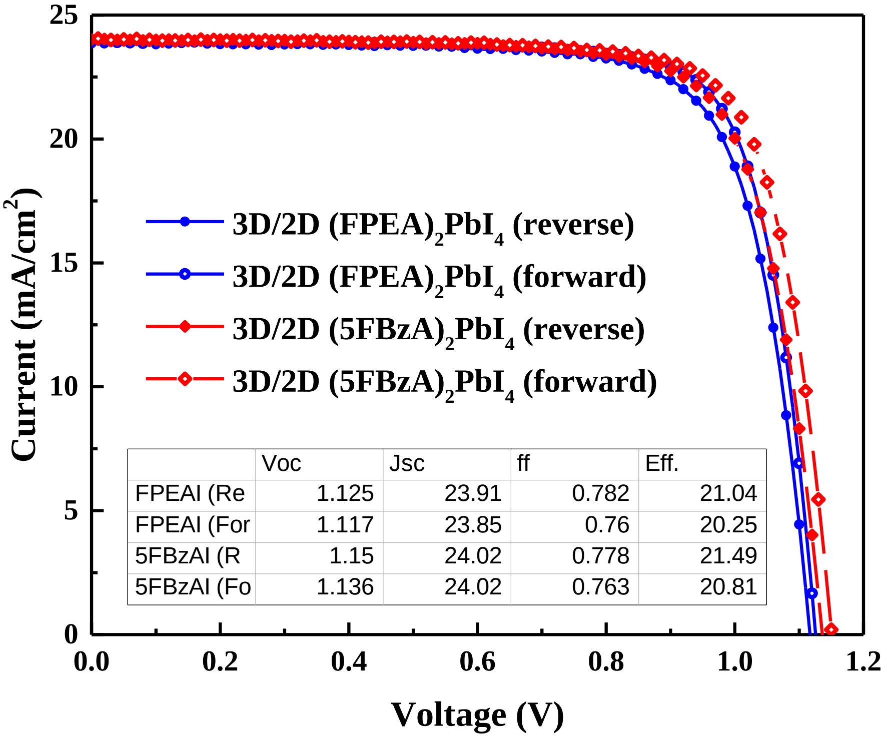

	Voc	Jsc	ff	Eff.
FPEAI (Re	1.125	23.91	0.782	21.04
FPEAI (For	1.117	23.85	0.76	20.25
5FBzAI (R	1.15	24.02	0.778	21.49
5FBzAI (Fo	1.136	24.02	0.763	20.81

Figure S1. Hysteresis of PSCs device from reverse and forward scan at a scan rate of 50 mV s^{-1} (Voc in V, Jsc in mA·cm^{-2}, and Eff in %).

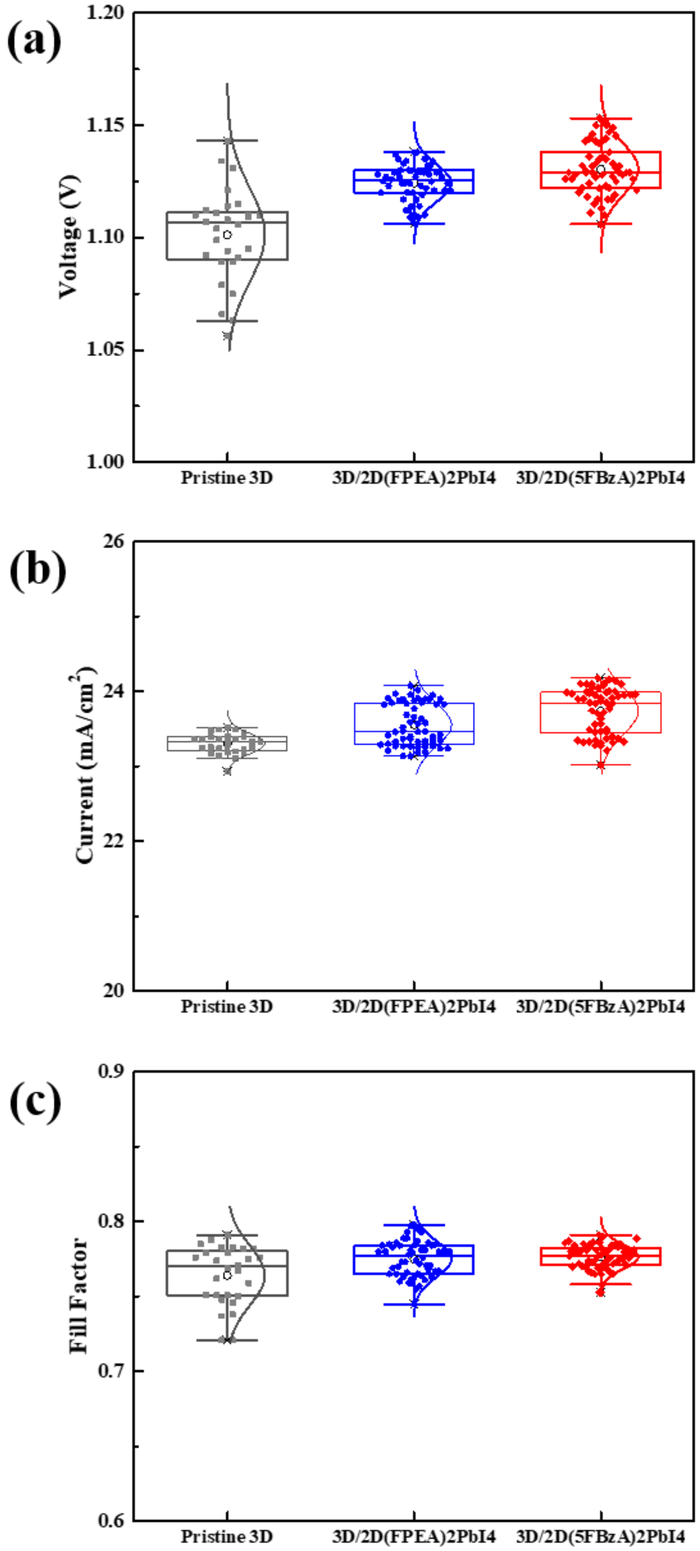

Figure S2. Histogram of photovoltaic parameters (Voc, Jsc, and FF) of perovskite solar cells with pristine 3D and 2D (FPEAI and 5FBzAI) treatment.

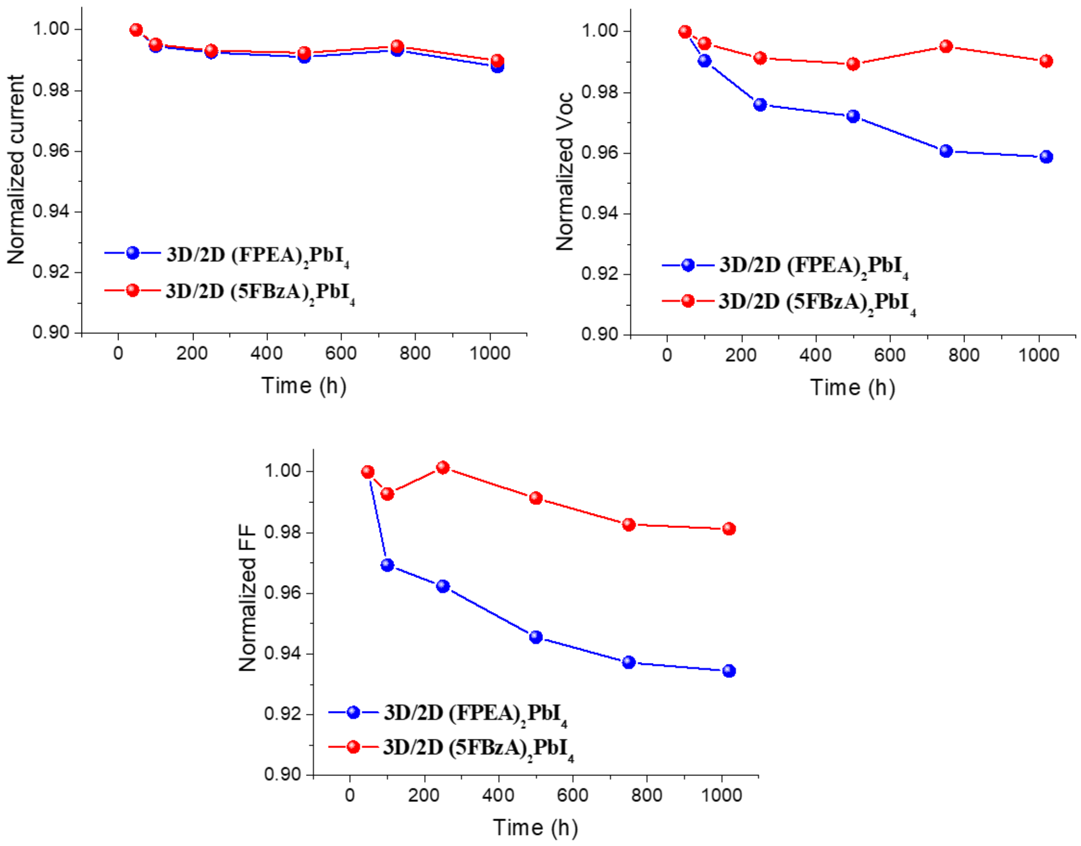

Figure S3. Evolution of the photovoltaic parameters during stability test for cells containing a bilayer structure of 3D/2D employing FPEAI and 5FBzAI cations, normalized at 50h of test.

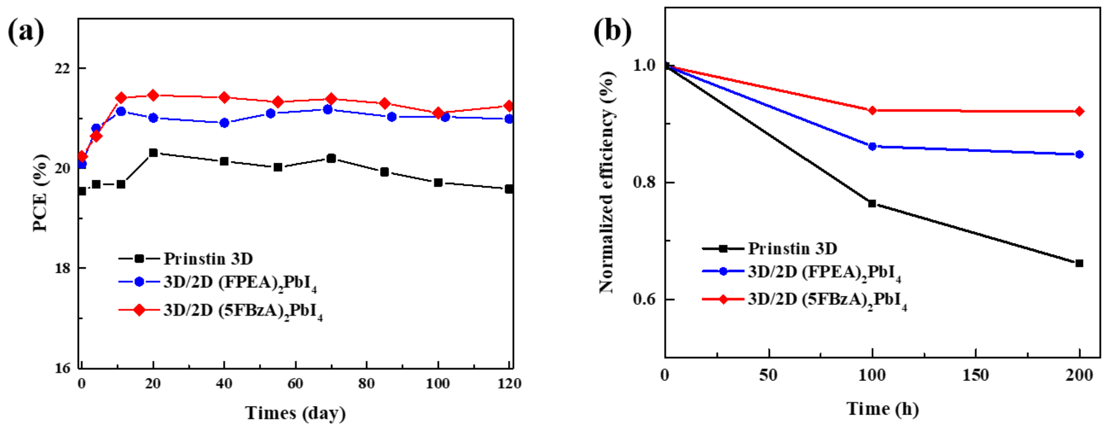

Figure S4. Long-term stability of pristine 3D and 3D/2D treatment perovskite device. a) The devices were stored under dark, in air atmosphere, with controlled relative humidity (10-15 %), b) the devices were store under dark with higher humidity (60%), and measured in ambient atmosphere

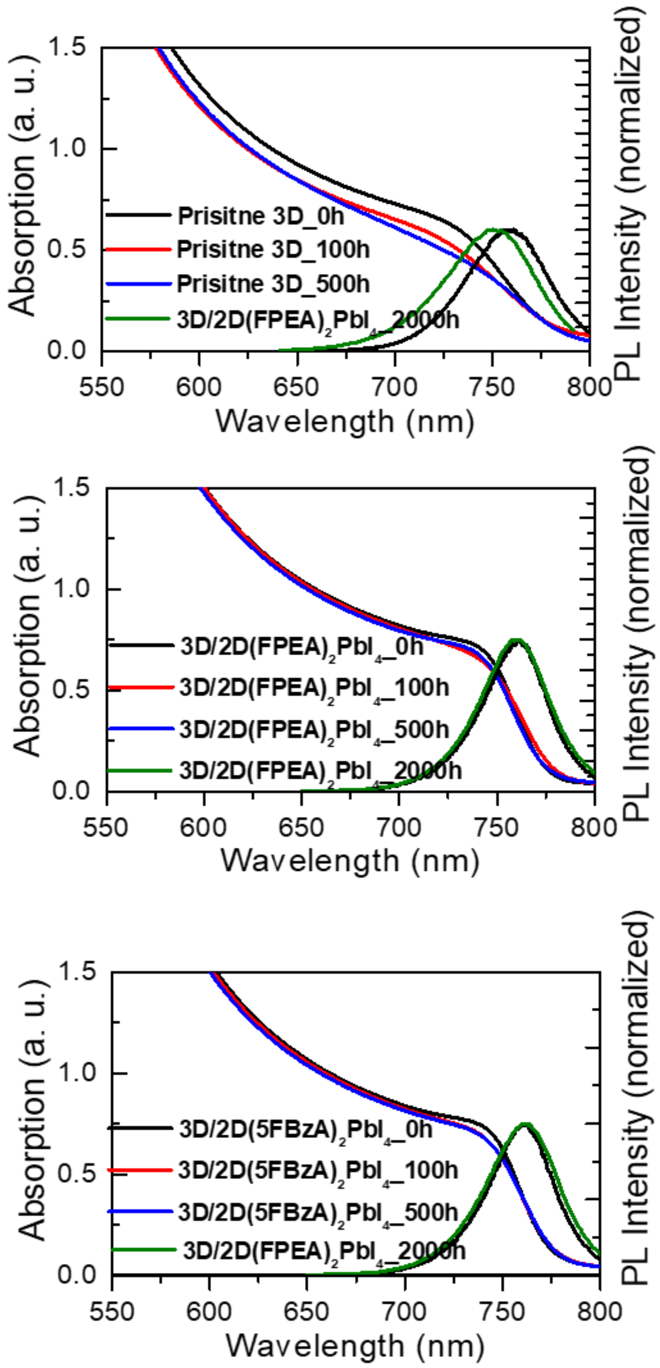

Figure S5. Absorption and photoluminescence spectra of pristine 3D and 3D/2D treated perovskite solar cells measured at different aging times.

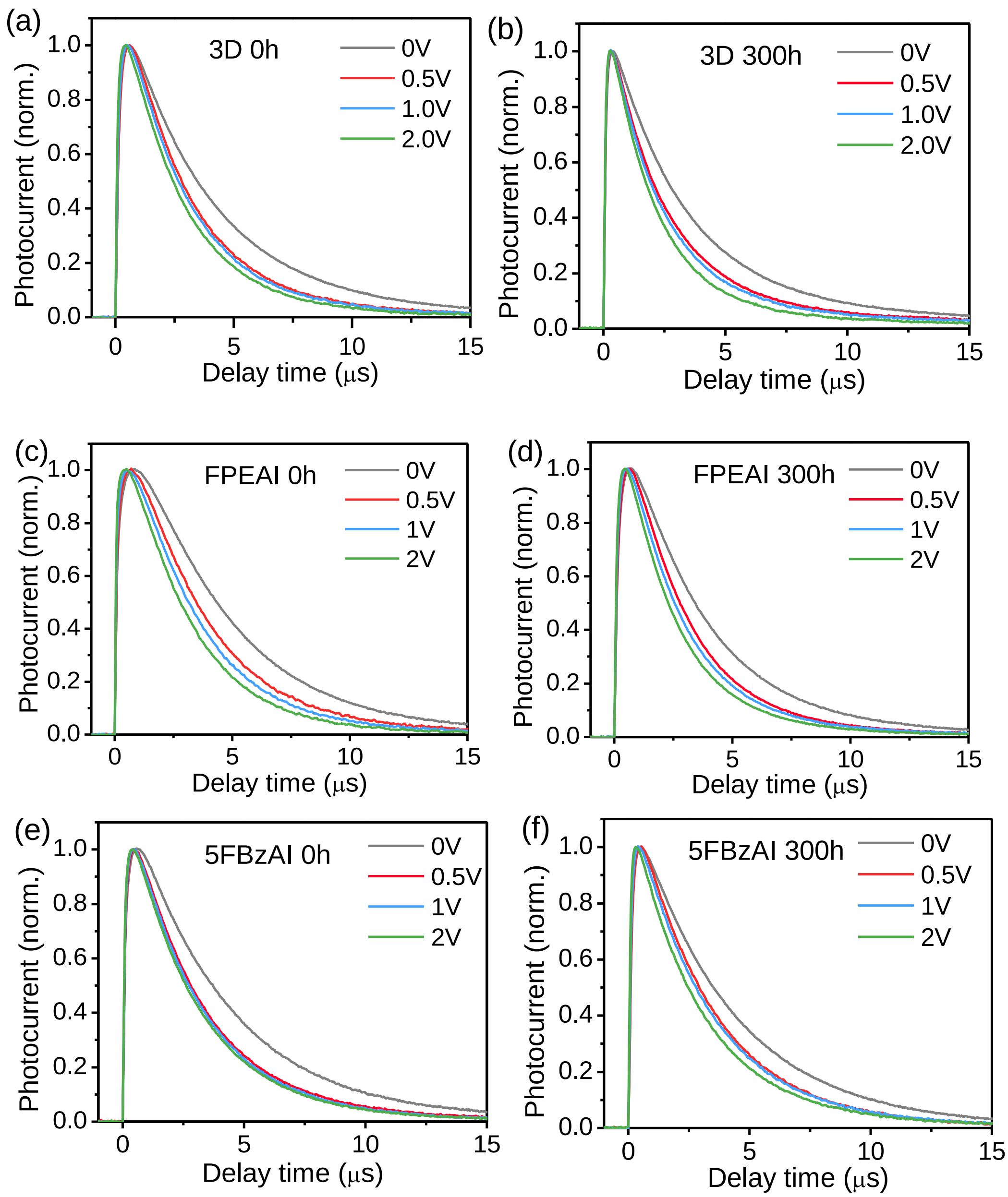

Figure S6. Transient photocurrent kinetics of the fresh (a, c, e) and aged for 300h (b, d, f) 3D (a, b), 3D/2D $(FPEA)_2PbI_4$ (c, d) and 3D/2D $(5FBzA)_2PbI_4$ (e, f) perovskite solar cells at different applied voltages.

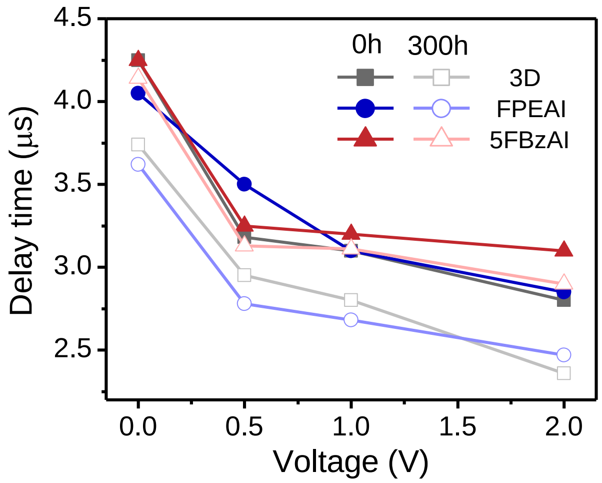

Figure S7. Transient photocurrent kinetics of the fresh and aged 3D, 3D/2D $(FPEA)_2PbI_4$ and 3D/2D $(5FBzA)_2PbI_4$ perovskite solar cells at different applied voltages.

Table S1. Crystal data and structure refinement for $(5FBzA)_2PbI_4$.

Identification code	$(5FBzA)_2PbI_4$
Empirical formula	$C_7H_5F_5I_2NPb_{0.5}$
Formula weight	555.51
Temperature/K	300.22
Crystal system	orthorhombic
Space group	Pbca
a/Å	8.6249(3)
b/Å	8.5384(3)
c/Å	35.2776(12)
α/°	90
β/°	90
γ/°	90
Volume/Å^3	2597.94(16)
Z	8
ρ_{calc}g/cm^3	2.841
μ/mm^{-1}	11.323
F(000)	1968.0
Crystal size/mm^3	0.14 × 0.04 × 0.03
Radiation	MoKα (λ = 0.71073)
2Θ range for data collection/°	4.618 to 65.936
Index ranges	$-12 \leq h \leq 13$, $-12 \leq k \leq 13$, $-54 \leq l \leq 40$

Reflections collected	25587
Independent reflections	4767 [R_{int} = 0.0351, R_{sigma} = 0.0281]
Data/restraints/parameters	4767/72/143
Goodness-of-fit on F^2	1.233
Final R indexes [I>=2σ (I)]	R_1 = 0.0568, wR_2 = 0.1181
Final R indexes [all data]	R_1 = 0.0807, wR_2 = 0.1249
Largest diff. peak/hole / e Å^{-3}	1.09/-2.87

Table S2. Summary of the fitting parameters for the PL decay of the pristine 3D, 3D/2D-FPEAI and 3D/2D-5FBzAI perovskite films.

Perovskite	A_1, %	τ_1, ns	A_2, %	τ_2, ns
3D (0h)	21	26	79	940
3D (50h)	34	21	66	480
3D (500h)	45	22	55	172
3D/2D$(FPEA)_2PbI_4$ (0h)	28	29	72	772
3D/2D$(FPEA)_2PbI_4$ (50h)	38	32	62	577
3D/2D$(FPEA)_2PbI_4$ (500h)	49	36	51	400
3D/2D$(5FBzA)_2PbI_4$ (0h)	18	46	82	938
3D/2D$(5FBzA)_2PbI_4$ (50h)	28	46	72	780
3D/2D$(5FBzA)_2PbI_4$ (500h)	30	47	70	568

Table S3. Surface binding energy of 2D perovskite and $FAPbI_3$ obtained from calculations in Note 1 (PBE-D3 with 550 eV cutoff and 3x3x1 k-point mesh. Negative value means not binding)

	FPEA (non-tilt)	5FPEA
E_{bind} (eV)	-0.704	-0.603
E_I	0.235	**-0.245**
E_{LE}	0.469	0.848

Table S4. Lattice parameter and lattice mismatch of 2D perovskite and $FAPbI_3$ in a 3D/2D stack structure.

	FPEA	5FPEA	$FAPbI_3$
a (Å)	12.22	12.14	12.81
b (Å)	12.22	12.14	12.53
mismatch in a	-4.61%	-5.23%	
mismatch in b	-2.47%	-3.11%	

Note S1. The binding energy (E_{bind}) of the 2D-3D bilayer was calculated according to the following equation:

$$E_{bind} = -(E_{2D+3D} - E_{2D} - E_{3D}) \qquad \text{Eq.1}$$

Where E_X is the energy associated to system X. Note that the interface was built based on $FAPbI_3$ lattice parameters. However the employed 3D material contains Cs and MA cations, which are smaller in size, and this leads to a considerably lattice mismatch in both systems producing negative values for the surface binding energy (-0.704 and -0.603 eV for FPEA and 5FPEA respectively). Nevertheless, 5FPEA shows larger binding energy indicating a stronger interaction at the interface. The lattice constant and mismatch for each structure is shown in Table 4. To evaluate the "chemical interaction" between 2D and 3D perovskite, we divided the binding energy into two parts: on one side the energy required to expand the lattice of 2D perovskite (E_{LE}) and secondly the interaction energy between 3D and 2D layers (E_I).

$$E_{bind} = -\left[\left(E_{2D+3D} - E_{2D,e} - E_{3D}\right) + \left(E_{2D,e} - E_{2D}\right)\right] = -[E_I + E_{LE}] \qquad \text{Eq. 2}$$

From Table S3, we can see that the interaction energy is much stronger (more negative) for 5FPEA, which may be attributed to halogen···halogen interaction between F and I.
